# Photoinduced granulomatous drug reaction related to lamotrigine

**DOI:** 10.1016/j.jdcr.2026.03.026

**Published:** 2026-03-20

**Authors:** Johanna Clerc, François Aubin, Benjamin Cretin, Christine Devalland

**Affiliations:** a Department of Pathology, Hôpital Nord Franche-Comté, Belfort, France; b Department of Dermatology, Centre Hospitalier Universitaire, Besançon, France; c Department of Neurology, Hôpitaux Universitaires de Strasbourg, Strasbourg, France

**Keywords:** drug eruption, granulomas, lamotrigine, phototoxicity

## Introduction

Lamotrigine is an aromatic antiepileptic drug used for treatment of epilepsy and bipolar disorders. Cutaneous side effects related to this molecule are frequent and concern 8.4% of patients, including 0.04% of toxic epidermal necrolysis. Other reported drug reactions include maculopapular exanthem, urticaria, drug rash with eosinophilia and systemic symptoms, acute generalized pustulosis, and fixed drug eruption.^1^ We report the first case of photoinduced granulomatous drug rash related to lamotrigine.

## Case report

**Fig 1:**
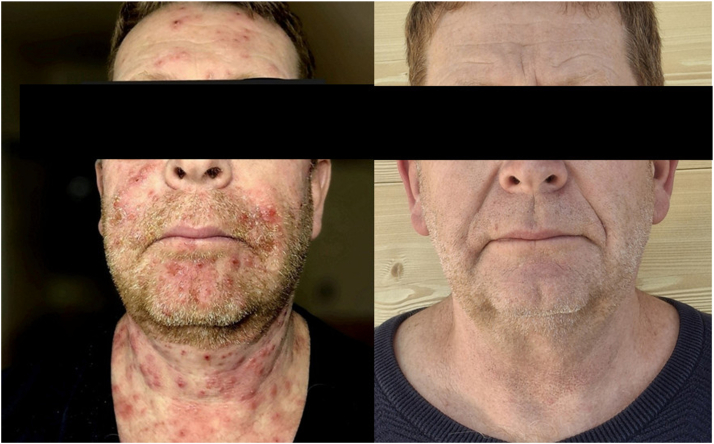
Clinical presentation, on the left, papulo-nodular and pustular eruption of photoexposed areas, on the right, complete clearance of lesions within less than one month after switching antiepileptic treatment.

A 50-year-old male with a Fitzpatrick skin type II was treated with lamotrigine as monotherapy for comitial seizures caused by an inoperable cerebral cavernous angioma. Treatment was introduced, stopped the comitial seizures. He developed a photodistributed rash with flare aggravations during intense sun exposure a few weeks after. Four years later, the skin lesions were permanent, characterized by an erythematous papulo-nodular and pustular rash only involving the face, neck, and nape of the neck, without mucosal involvement (Fig 1). The lesions were not pruritic and did not leave atrophic scars.

He did not present any systemic symptoms.

Completed blood examination showed normal values with no eosinophilia, no hypergammaglobulinemia, no renal and liver dysfunction, and an antinuclear antibody titer of 1:80.

**Fig 2:**
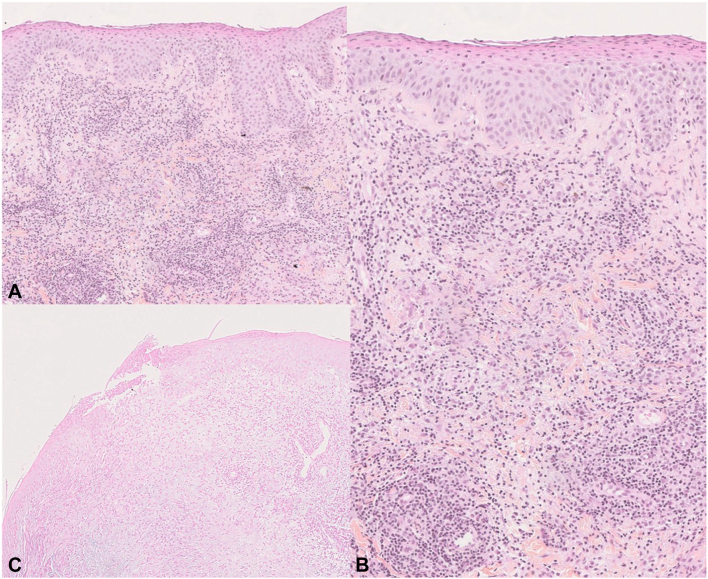
Hematoxylin–eosin–saffron staining showing perivascular and interstitial granulomatous inflammation of the entire dermis (**A,** ×10). Spongiosis, slight interface dermatitis, polymorphic infiltrate (histiocytes, few giant cells, lymphocytes, plasma cells, eosinophilic and neutrophilic cells), no necrobiosis (**B,** ×20). Alcian blue staining, very slight mucin deposition (**C,** ×10).

Histologically, the skin biopsy slides stained with hematoxylin–eosin–saffron (Fig 2, *A* and *B*) revealed a diffuse granulomatous infiltration of the entire dermis, perivascular and interstitial, with focal fragmentation of collagen and elastic fibers, very slight mucin deposition (Fig 2, *C*) and no necrobiosis. This infiltrate was polymorphic with histiocytes, few giant cells, lymphocytes, plasma cells, and eosinophilic and neutrophilic cells.

Lymphocytes showed prominent markers for CD4, CD8, rare CD30, and moderate nuclear atypia. The epidermis was spongiotic and pustular, with vacuolar interface dermatitis, but no necrotic keratinocytes.

There was no elastophagy with Orcein staining, no vasculitis, and no panniculitis. Periodic acid–Schiff, Grocott, gram, and Ziehl staining results were negative.

We observed only partial improvement of symptoms with daily application of ultra-high strength topical steroid (clobetasol propionate, 0.05%) and strict photoprotection. Skin lesions completely regressed when lamotrigine were substituted by sodium valproate, without recurrence with a follow-up of 12 months.

## Discussion

Granulomatous lesions are immune reactions to an endogenous or exogenous agent, initiated by antigen-presenting cells that activate the secretion of interleukin-2 and interferon-gamma by T lymphocytes. T lymphocytes then activate macrophages, which transform into epithelioid histiocytes and giant cells.^2^

Granulomatous drug reactions are classified into several histological subtypes: interstitial granulomatous drug reaction (IGDR), drug-induced granuloma annulare (GA), drug-induced elastolytic actinic giant cells granuloma (EAGCG), drug-induced accelerated rheumatoid nodulosis, drug-induced sarcoidosis, cutaneous granulomatous reactions at injection sites, and photoinduced granulomatous drug reaction.^2^^,^^3^

Granulomatous drug reactions have been reported more frequently since the use of targeted therapies and immunotherapy in oncology^4^ and the use of biological therapeutics in chronic inflammatory diseases.

Systemic granulomatous involvement is rare in IGDR.^2^ There is limited information on this subject for photoinduced granulomatous drug reactions. Our patient had no systemic involvement, so as the rare reported cases.^5^^,^^6^ However, cases of acute granulomatous nephritis and colitis on lamotrigine have been reported, without cutaneous involvement.^7^

The pathophysiology of IGDR is poorly understood. The drug probably induces immunogenicity changes of the dermis that cause granulomatosis.^3^

The relationship between granulomatous drug reaction and photoexposure is unknown, and only very few cases of photoinduced granulomatous drug reactions have been reported, except EAGCG.^5^^,^^6^

The photodistributed nature of the lesions raises questions about an associated photoallergic or phototoxic phenomenon. Mechanisms of photoallergy and phototoxicity are different and often difficult to distinguish clinically. Histologically, photoallergy results in a lympho-histiocytic spongiotic dermatitis close to allergic contact eczema, whereas phototoxicity results in a lichenoid dermatitis with necrotic keratinocytes and a lymphocytic and neutrophilic inflammatory infiltrate.^8^

A case of toxic epidermal necrolysis of photoexposed areas related to lamotrigine has been reported.^9^ Two hypotheses have been suggested: (1) ultra-violet radiation leads to an increased deposition of immune complexes in the skin or (2) phototoxic metabolites of the drug accumulate in the skin and undergo slow photodegradation, releasing chloride anions and free radicals and leading to phototoxicity.^10^

This granulomatous drug reaction in photoexposed areas is therefore likely multifactorial. It may involve modification of dermal immunogenicity related to the drug and chronic photoexposure, a possible phototoxic metabolite of lamotrigine, and a combined photoallergic mechanism, as suggested by the presence of spongiosis, mild interface dermatitis, and the absence of epidermal necrosis.

In our case, the granulomas were nonsarcoid. The granulomatous infiltrate was highly polymorphic with poor necrobiosis, which excludes GA. The absence of elastolysis and elastophagy ruled out EAGCG. Angiocentrism and polymorphism of the granulomas with eosinophilic cells could suggest granuloma faciale, but clinical presentation and lack of hypercellularity were inconsistent. The polymorphism of the granulomas was also inconsistent with connective tissue disease or lymphoma despite the nuclear atypia of the lymphocytes.

Once infectious and lymphomatous granulomatosis have been eliminated along with histologically classical entities (GA, EAGCG, sarcoidosis, accelerated rheumatoid nodulosis), the diagnosis of IGDR must be considered. Lesion onset varies from a few weeks to a few months after drug intake, as does the clearance of symptoms when the treatment is stopped.^2^ This makes the diagnosis of granulomatous drug reactions challenging, especially in cases involving polymedication, and anatomo-clinical correlation is essential.

Drug-induced GA, EAGCG, sarcoidosis, and accelerated rheumatoid nodulosis do not appear to have histological criteria pointing to a drug origin.^3^^,^^5^^,^^6^ Only the clinical investigation may lead to a drug origin suspicion.

In contrast, IGDR is a polymorphic entity that presents as a set of histological criteria suggestive of a drug reaction, including a polymorphic inflammatory infiltrate with eosinophilic cells, atypical T lymphocytes, interface dermatitis, and epidermic remodeling. Despite its name, it is often not exclusively interstitial and may also be nodular or perivascular, with occasional necrobiosis, elastolysis, and elastophagy. IGDR is a challenging diagnosis and probably underdiagnosed.

## Conflicts of interest

None disclosed.
